# Development and initial validation of the bronchiectasis exacerbation and symptom tool (BEST)

**DOI:** 10.1186/s12931-019-1272-y

**Published:** 2020-01-13

**Authors:** Amaia Artaraz, Megan L. Crichton, Simon Finch, Hani Abo-Leyah, Pieter Goeminne, Stefano Aliberti, Thomas Fardon, James D. Chalmers

**Affiliations:** 1 Department of Respiratory Medicine, Galdakao-Usansolo Hospital, Galdakao, Bizkaia Spain; 2 Scottish Centre for Respiratory Research, Ninewells Hospital and Medical School, Division of Molecular and Clinical Medicine, University of Dundee, DD1 9SY Dundee, Scotland; 3 AZ Nikolaas, Sint-Niklaas, Belgium; 4 Department of Pathophysiology and Transplantation, University of Milan, Milan, Italy; 5 Fondazione IRCCS Ca’ Granda Ospedale Maggiore Policlinico, Respiratory Unit and Cystic Fibrosis Adult Center, Milan, Italy

**Keywords:** Bronchiectasis, Exacerbations, Unreported exacerbations, Symptom diary

## Abstract

**Background:**

Recurrent bronchiectasis exacerbations are related to deterioration of lung function, progression of the disease, impairment of quality of life, and to an increased mortality. Improved detection of exacerbations has been accomplished in chronic obstructive pulmonary disease through the use of patient completed diaries. These tools may enhance exacerbation reporting and identification. The aim of this study was to develop a novel symptom diary for bronchiectasis symptom burden and detection of exacerbations, named the BEST diary.

**Methods:**

Prospective observational study of patients with bronchiectasis conducted at Ninewells Hospital, Dundee. We included patients with confirmed bronchiectasis by computed tomography, who were symptomatic and had at least 1 documented exacerbation of bronchiectasis in the previous 12 months to participate. Symptoms were recorded daily in a diary incorporating cough, sputum volume, sputum colour, dyspnoea, fatigue and systemic disturbance scored from 0 to 26.

**Results:**

Twenty-one patients were included in the study. We identified 29 reported (treated exacerbations) and 23 unreported (untreated) exacerbations over 6-month follow-up. The BEST diary score showed a good correlation with the established and validated questionnaires and measures of health status (COPD Assessment Test, *r* = 0.61, *p* = 0.0037, Leicester Cough Questionnaire, *r* = − 0.52,*p* = 0.0015, St Georges Respiratory Questionnaire, *r* = 0.61,*p* < 0.0001 and 6 min walk test, *r* = − 0.46,*p* = 0.037). The mean BEST score at baseline was 7.1 points (SD 2.2). The peak symptom score during exacerbation was a mean of 16.4 (3.1), and the change from baseline to exacerbation was a mean of 9.1 points (SD 2.5). Mean duration of exacerbations based on time for a return to baseline symptoms was 15.3 days (SD 5.7). A minimum clinically important difference of 4 points is proposed.

**Conclusions:**

The BEST symptom diary has shown concurrent validity with current health questionnaires and is responsive at onset and recovery from exacerbation. The BEST diary may be useful to detect and characterise exacerbations in bronchiectasis clinical trials.

## Introduction

Bronchiectasis is a chronic respiratory disease defined by abnormal and irreversible dilatation of the bronchi [1]. The disease can be caused by many different aetiologies and it is clinically characterised by a variety of symptoms, including cough, sputum production and airway infection, and can often present with recurrent exacerbations [2]. According to a recent international consensus of experts, exacerbations are defined by an impairment of at least 3 or more baseline symptoms including cough, sputum volume and/or consistency, sputum purulence, dyspnoea and/or exercise tolerance, fatigue and/or malaise, haemoptysis for at least 48 h requiring a change in treatment [3]. Recurrent exacerbations are related to elevated systemic and airway inflammation, deterioration of lung function and progression of the disease [4–7]. As a consequence, exacerbations represent one of the main causes of healthcare costs in bronchiectasis. Some patients experience very frequent exacerbations and these have been described a phenotype with a higher morbidity and mortality [5]. Reduction of exacerbations is therefore, the key goal of most therapeutic interventions [8].

Exacerbations have been the primary endpoints in the majority of therapeutic phase 3 clinical trials in bronchiectasis [9–11]. Unfortunately the results of recent trials investigating inhaled antibiotics and inhaled mucoactive drugs have given inconsistent results. The RESPIRE trials for example, recruited patients with a history of at least 2 exacerbations in the previous year, with the expectation they would experience a similar rate of exacerbations during follow-up. The rates in the four RESPIRE studies in the placebo groups of 0.8, 1, 0.7 and 0.7 exacerbations per patient per year made it extremely difficult to demonstrate a meaningful treatment effect [12]. Under-reporting and under-detection of exacerbations may play a role in this phenomenon.

Improved reporting and detection of exacerbations, as well as greater understanding of the natural history of exacerbations has been achieved in Chronic obstructive pulmonary disease through the use of patient completed diaries [13]. These tools may enhance exacerbation reporting and thereby identification [14], leading to more efficient treatment when indicated. It has been suggested that 2/3 of COPD exacerbations are not reported to health care professionals [13, 15]. These are important events to recognize using diaries because unreported exacerbations have been linked to a greater risk of hospitalization, lung function deterioration, particularly forced expiratory volume in 1 s (FEV_1_), and worse St George’s respiratory questionnaire (SGRQ) scores than treated exacerbations [15, 16].

The Exacerbations of chronic obstructive pulmonary disease tool (EXACT) can detect the frequency, duration and severity of exacerbations in patients with COPD [13, 17].

The symptoms of COPD and COPD exacerbations are similar but not identical to those of bronchiectasis. Development of a symptom diary specific for bronchiectasis could provide substantial benefits for bronchiectasis research by identifying events in clinical trials and providing detailed insight to bronchiectasis exacerbations, including unreported events.

This manuscript describes the development and initial validation of a novel symptom diary, the BEST diary (Bronchiectasis Exacerbation and Symptoms Tool) to detect bronchiectasis exacerbations.

## Methods

This was a prospective observational study of adults with bronchiectasis conducted at a specialist bronchiectasis centre at Ninewells Hospital, Dundee, UK. The study was approved by the local research ethics committee (13/ES/0062) and all patients gave written informed consent to participate. Inclusion criteria required high resolution CT (computerized tomography) confirmed bronchiectasis; that bronchiectasis was clinically significant with at least one of daily cough, sputum production or a history of recurrent respiratory tract infections and patients had to have at least one documented exacerbation of bronchiectasis in the previous 12 months to participate. Exclusion criteria were age < 18 years, a primary diagnosis of another respiratory condition (including co-existing COPD and asthma) and cystic fibrosis. Patients had to be clinically stable at enrolment for a period of at least 4 weeks without treatment with antibiotics or corticosteroids for an exacerbation. Receiving maintenance oral or inhaled antibiotics was permitted.

### Development of a symptom diary

The study objective was to develop a symptom diary that measures day to day changes in patient symptoms but also accurately detects exacerbations. The study was conducted in parallel with the development of the consensus definition of exacerbations by EMBARC (European Multicentre Bronchiectasis Audit and Research Collaboration). and the Bronchiectasis Research Registry (EMBARC/Bronchiectasis Research Registry- BRR) [3]. As previously reported, the authors conducted a systematic review of definitions of exacerbation used in previous studies in bronchiectasis. The results of this systematic review are reported in the EMBARC/BRR publication but were also used for the development of this diary. Further information about the results of the systematic review are provided in the Additional file 1. As described in the EMBARC/BRR publication, the symptoms most frequently used in prior definitions, and rated by Delphi process as most important were sputum volume, cough, sputum colour, dyspnoea, fatigue and systemic disturbance [3]. Patient self-report of an exacerbation was also considered important in the Delphi process. Based on these results and interviews with patients who also identified the same critical symptoms [18], we designed a daily diary that appropriately rated these symptoms. The diary was modified with feedback from patients in terms of wording and clarity of meaning until a final version was completed and deployed in the study. For rating dyspnoea we incorporated the existing MRC dyspnoea score as it is validated in bronchiectasis. The sputum colour domain followed the previously published scale by *Murray* et al which rates sputum from 1 to 4 ranging from clear to dark green, with additional domains of no sputum (0) and haemoptysis [5, 19]. The topic “systemic symptoms” had little meaning to patients and was modified to cold and flu symptoms. Quantification of sputum volume using millilitres was also regarded as difficult for most patients and so equivalent volumes using teaspoon/tablespoon, egg-cup and cup volumes were used. A teaspoon is equivalent to 5 ml, a tablespoon is equivalent to 15 ml, egg-cup 45 ml and cup volume is approximately 250 ml. The final diary is shown in Table 1. The maximum score was 26.

**Table 1:** The BEST (Bronchiectasis exacerbation and symptom tool) diary card

	BREATHLESSNESS		FATIGUE
0	None	0	I do not feel tired
1	Breathlessness when hurrying or walking up a slight hill	1	I feel a little tired
2	Have to walk slowly on level ground or stop for breath after a few minutes on level ground	2	I feel tired but can still do the things I would like to do
3	Can walk less than 100 m or a few minutes on level ground before having to stop	3	Tiredness is stopping me from doing some things I want to do
4	Breathless when washing or dressing	4	I am so tired I am unable to carry out my usual daily activities
	SPUTUM VOLUME		SPUTUM COLOUR
0	No sputum	0	No sputum
1	Less than a teaspoon	1	White
2	Teaspoon to an eggcup	2	Yellow
3	Egg-cup to a cup	3	Green
4	More than a cup	4	Dark Green
5	–	5	Blood stained
	COUGH		COLD AND FLU SYMPTOMS
0	None	0	None
1	Mild	1	Sore throat, sore muscles, or runny nose
2	Moderate	2	Fever/high temperature or shivers
3	Severe	3	–
4	Very severe	4	–
5	–	5	I feel like a have an infection

### Study design

Patients were asked to complete the diary every day for a period of up to 6 months while clinically stable and then when they experienced an exacerbation. The patients were asked to contact the investigators when they experienced an exacerbation so that treatment could be commenced by the study team using a standard 14-day course of antibiotics. The tool was not used to trigger contact with the study team.

At the baseline visit patients were issued with the diary and provided with education on its completion. Paper diaries were used to avoid any bias in this population towards those familiar with mobile technologies. Patients completed the SGRQ, the COPD assessment test (CAT) and the Leicester cough questionnaire (LCQ) at the baseline visit. Patient enrolment took place in 2014 prior to the development of the disease specific tools such as the Quality of life bronchiectasis questionnaire (QoL-B) or the Bronchiectasis Health Questionnaire (BHQ) [20, 21]. Patients underwent spirometry and performed a 6-min walk test (6MWT) to evaluate convergent validity with these measures of lung function and functional status respectively. The assessments were repeated at the start and end of each exacerbation and finally at the end of study.

### Validation of the symptom diary

We addressed firstly whether the symptom diary accurately measured symptom burden in patients with bronchiectasis by demonstrating its convergent validity with existing symptoms and quality of life (QOL) measures. We hypothesised that the baseline symptom diary score would correlate with the SGRQ total and symptom scores, the LCQ, the COPD CAT and 6MWT. We next analysed the dynamic changes in bronchiectasis symptoms over time. We hypothesised that symptom scores would increase during periods of reported exacerbations. To detect unreported exacerbations we required an increase of at least 3 points in symptoms for a minimum of 48 h based on the EMBARC/BRR definition of exacerbation [3]. An unreported exacerbation by definition was associated with sustained increase in symptoms but did not lead to a medical review or antibiotic treatment.

### Calculation of the minimum clinically important difference

We estimate a provisional minimum clinically important difference for the BEST tool. As there is no single accepted method of determining the minimal clinical important difference (MCID) we used 3 different approaches. First, the distribution based method using ½ the standard deviation of the scores for all participants. Second, if a correlation coefficient of > 0.3 was achieved for the relationship with scores which had an established MCID (such as the SGRQ, CAT and LCQ) we would use these as an “anchor” to estimate the change in BEST score that equated to a clinically meaningful change in the other tool. Finally as a reported exacerbation is a clinically meaningful change in symptoms, we would consider the change in BEST at exacerbation onset as a potential MCID [22].

### Statistical analysis

Statistical analysis was performed using Graphpad Prism v6 and SPSS version 22 (IBM, USA). Categorical variables are presented by frequencies and percentages while continuous variables are presented as mean and standard deviation (SD) or median and interquartile range (IQR) when data are not distributed normally. The relationship between the diary scores and quality of life/symptom measures such as SGRQ, CAT, LCQ and 6MWT were performed by linear regression. The regression equation was used to calculate the change in BEST score that would equate to the MCID scores of 4 points in the SGRQ, 2 points in the CAT and 1.3 points in the LCQ [22, 23]. Students T-test was used to compare means between different time points such as stability and exacerbation. Stability of symptoms within individual patients was evaluated with the within subject standard deviation and the coefficient of variation. To establish patients’ baseline level of symptoms we took the median value in the week prior to an event (or in the first week of the study). As this was a pilot study, no formal sample size calculation was performed and the objective was to enrol 20 patients to get initial experience with the tool. For all analyses a *p*-value < 0.05 was considered statistically significant.

## Results

### Description of the patients

Twenty-one patients were included. All patients had idiopathic or post-infective bronchiectasis. The characteristics of the patients are shown in Table 2. Patients had predominantly moderate to severe bronchiectasis and in common with most bronchiectasis populations, were elderly and female. As the study enrolled patients with a history of exacerbations the mean exacerbation and use of prophylactic therapies such as macrolides was high in this cohort.

**Table 2:** Characteristics of the included patients

Characteristic	Mean (sd) or n (%)
Age	67.5 (7.4)
Female sex	16 (76.2%)
Comorbidities	
Cardiovascular disease	1 (4.8%)
Osteoporosis	4 (19.1%)
Anxiety/depression	3 (14.3%)
Diabetes	2 (9.5%)
FEV1	1.76 (0.60)
FEV1% predicted	78.7% (21.7)
FVC	2.74 (0.87)
Bronchiectasis severity index	
Mild	4 (19.0%)
Moderate	10 (47.6%)
Severe	7 (33.3%)
Prior exacerbations	3.3 (2.0)
Treatment	
Inhaled corticosteroids	11 (52.4%)
Macrolides	12 (57.1%)
Inhaled antibiotics	1 (4.8%)
Baseline quality of life and functional status	
6 min walk distance	439 (104)
CAT score	19.1 (6.1)
SGRQ	41.5 (17.0)

Nineteen patients completed the diary daily for at least 3 months. One patient had an exacerbation within the first week of starting the diary and did not persist with completion following exacerbation resolution, while another patient completed 6 weeks without an exacerbation and then ceased to adhere consistent data entry. Baseline data for the BEST are therefore shown for *n* = 21 patients while dynamic changes over time are analysed for *N* = 19 who had available long term data.

### Validation of symptom diary through correlation with other measures of health status

**Fig. 1:**
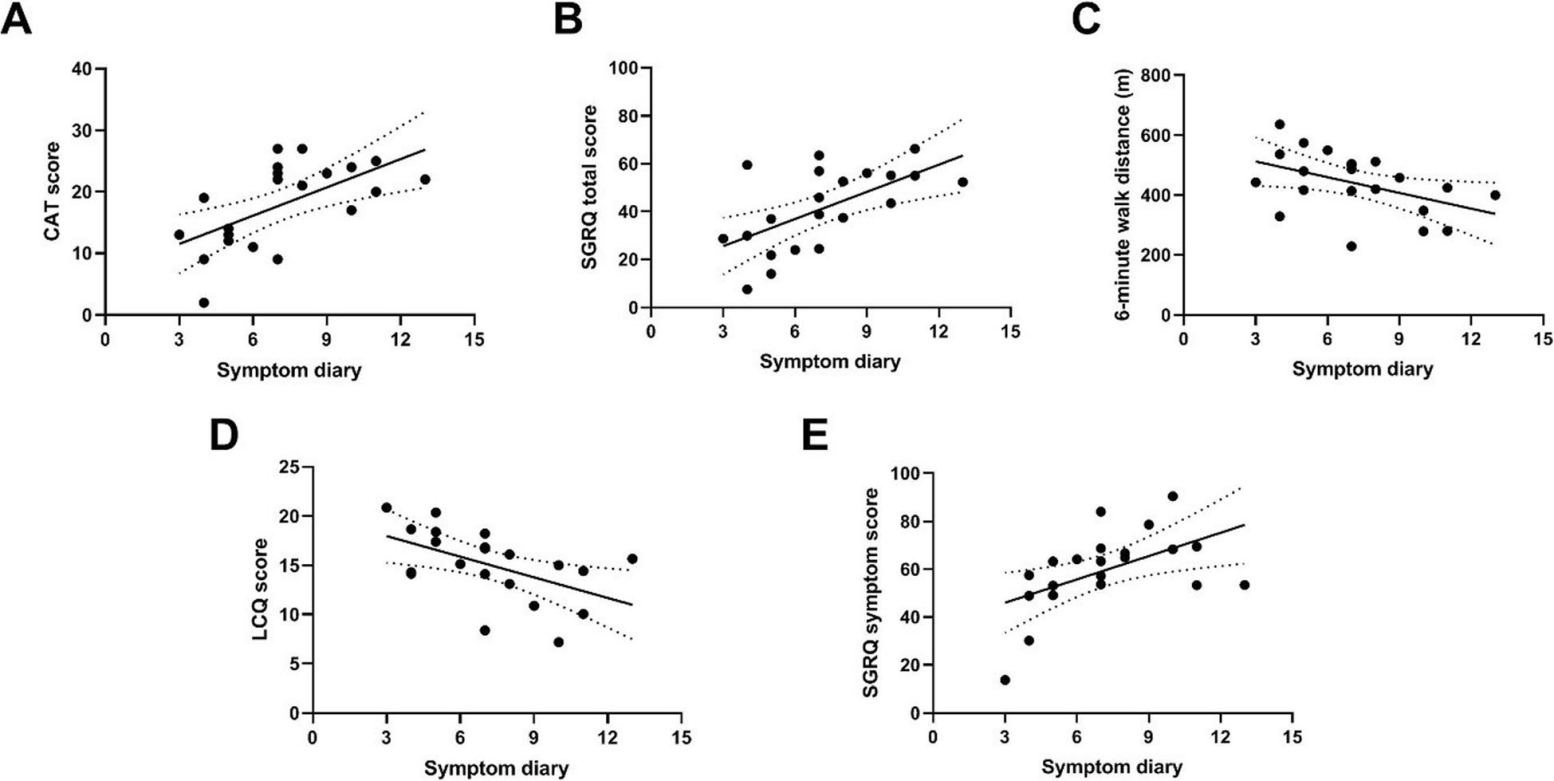
Correlations between the baseline BEST score and existing methods of evaluating symptoms and functional status **a**: CAT score, **b**: SGRQtotal score, **c**: 6-min walk distance, **d**: Leicester cough questionnaire (LCQ), **e**: Symptom domain of the SGRQ

At baseline, all patients completed the SGRQ, LCQ, CAT questionnaires and performed a 6MWT as measures of health status. The relationship between the baseline BEST score and the established measures of health status are shown in Fig. 1. The correlation with the CAT score (Fig. 1a) was *r* = 0.61, *p* = 0.0037. A significant correlation was also detected between the BEST score and the SGRQ total score (r = 0.61, *p* < 0.0001), Fig. 1b, 6MWT (*r* = − 0.46, *p* = 0.037,Fig. 1c), LCQ score (*r* = − 0.52,*p* = 0.015), Fig. 1d, and the symptom domain of the SGRQ (*r* = 0.52, *p* = 0.015),

A 4-point change in the SGRQ is regarded as clinically significant. The above correlation suggested that a change of 3.5 points in the BEST symptom diary was equivalent to a 4-point change in the SGRQ. For the LCQ, a 1.3-point change is regarded as clinically significant, and this correlated to a 4.95-point change in the BEST score. A clinically significant 2-point change in the CAT required a 4-point change in the BEST symptom diary. Taking a cut-off of 4 points, the change in BEST score had a sensitivity of 93% and specificity of 66% for detection of treated exacerbations. Raising the threshold to 5 points had a sensitivity of 86% and specificity of 81%. At 6 points the sensitivity declined to 79% with specificity of 82%.

### Dynamic changes in symptoms over time and detection of exacerbations

**Fig. 2:**
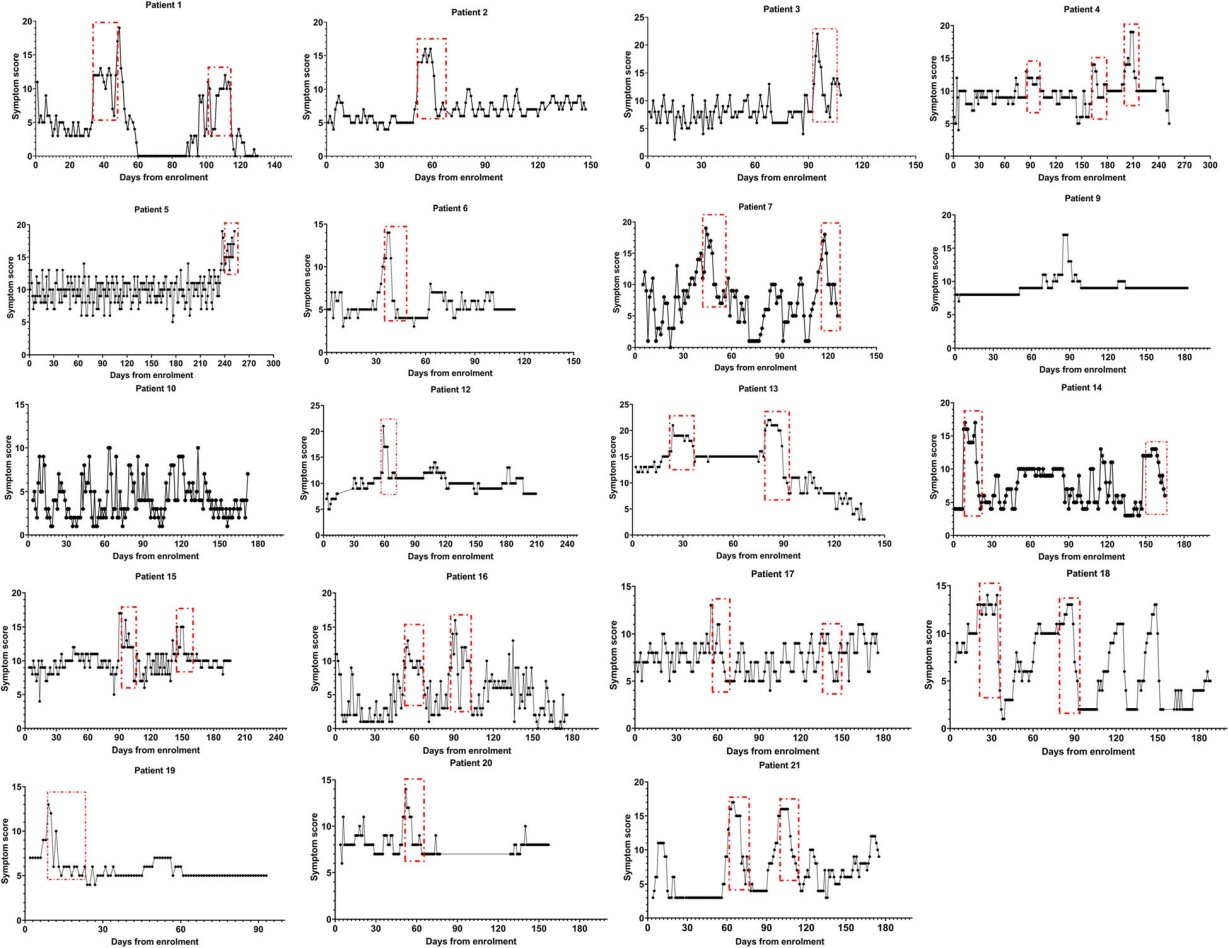
Individual patient dynamics during the study. The x-axis shows the time in the study which each point representing a single days score. The Y-axis shows the BEST score

Figure 2 below shows the dynamics of symptoms over time for 19 patients who were able to complete the diaries consistently for at least 3 months. In the figure below, the patient reported exacerbations are identified with a red box. 29 exacerbations in total were reported during the study.

The mean BEST score at baseline was 7.1 points (SD 2.2). Symptoms generally increased over several days prior to the diagnosis of an exacerbation. The mean score during this “pre-exacerbation” period was 11.3 points (SD 2.7) with a mean change of + 4 points (SD 1.8) (*p* < 0.0001 by paired t-test). The peak symptom score during exacerbation was a mean of 16.4 (3.1) (range 12–22). The change from baseline was a mean of 9.1 points (SD 2.5) with a range of 5 to 14. Interestingly 7 out of 29 exacerbations (24%) reported by the patients did not meet the EMBARC/BRR definition because of the presence of only 1 or 2 symptoms.

Based on the time taken for the symptom score to return to the baseline level post-exacerbation, symptoms remained elevated for 15.3 days after exacerbation diagnosis (SD 5.7). The range of recovery time was 5 days to 28 days.

### Unreported exacerbations and symptom variability

Based on a sustained increase in 3 points for at least 48 h, we identified 23 unreported exacerbations in 13 patients. No patients received antibiotic treatment during periods of unreported exacerbations. 8 patients had no unreported exacerbations. The mean change from baseline in an unreported exacerbation was 4.7 (SD 1.5, range 3–8 points). The mean duration of unreported exacerbations was 10 days (SD 3.8). Unreported exacerbations were therefore significantly milder and shorter than reported exacerbations. The end of the unreported exacerbation was considered when the score returned to pre-exacerbation baseline level. The sample size was too small to establish any relationships between unreported exacerbations and clinical outcomes.

We observed that some patients had relatively stable symptoms over time while others showed remarkable day to day variability in symptom scores. The coefficient of variation in symptom scores, excluding exacerbation periods, ranged from 16.1 to 97.8%. No relationship was observed between the co-efficient of variation or the within subject standard deviation and patient characteristics (Table 3).

**Table 3:** Differences between reported and unreported exacerbations. Onset time: number of days prior to commencing antibiotics

	REPORTED EXACERBATIONS	UNREPORTED EXACERBATIONS
Onset time (days)	4.8 (SD 3.5)	Not applicable
Change from baseline	9.1 (SD 2.5)	4.7 (SD 1.5)
Peak score	16.4 (SD 3.1)	11.1 (SD 3.0)
Duration	15.3 days	10 days

### Provisional minimum clinically important difference

The correlation with the SGRQ and CAT scores suggested an MCID of 4 points would be consistent with a clinically important change in other tools. Using the ½ standard deviation method to calculate the MCID suggested a smaller MCID of 2.2 points. Exacerbation onset was associated with a change of 4 points as described above. Based on the majority of data available we propose an initial MCID of 4 points.

## Discussion

The most relevant findings of our study were as follows: I) a newly developed symptom diary (hereafter referred to as the BEST tool) showed a good correlation with the established and widely validated questionnaires and measures of health status, including CAT, LCQ, SGRQ and 6MWT. II) Patients experienced a statistically significant increase in symptoms prior to reporting an exacerbation and the diary detected a large increase in symptoms during acute exacerbations requiring antibiotic treatment. III) After an exacerbation, symptoms remained elevated for 15.3 days (SD 5.7) before returning to the pre-exacerbation baseline level IV) 23 unreported exacerbations were identified in our cohort. To the authors knowledge this is the first description of unreported exacerbations in bronchiectasis. These events were significantly shorter and milder than reported exacerbation events V) We report a provisional MCID for the BEST tool with a suggestion that a 4-point change may be a clinically meaningful change in symptoms. The BEST tool now requires broader validation in larger patient cohorts. The diary is simple to complete and can be completed on paper or is easily adaptable to an application for electronic devices.

A validated symptom diary could be useful in the detection of exacerbations in the context of clinical research studies as well as randomized clinical trials. To the best of our knowledge, this is the first study to validate a symptom diary in bronchiectasis patients. Our symptom diary showed a statistically significant correlation with widely validated health status measures [24]. During the study period 29 exacerbations were reported. In our study, BEST symptom diary enabled the measurement of day to day changes in symptomatic bronchiectasis patients and allowed the detection of the onset, peak and duration of exacerbations. While some studies have used quality of life tools such as the Quality of life bronchiectasis questionnaire to study the change in symptoms at the time of an exacerbation, these tools compare a measure of symptoms at one time point, often remote from the exacerbation by months, and a change at a single time point in the exacerbation which may be not be the peak of symptoms [20]. The QOL-B questionnaire has a 1 week recall period. Our data shows that symptoms are highly variable and dynamic in bronchiectasis patients. The earliest detectable change at the onset of an exacerbation was a mean 4-point deterioration in the score, but the peak of symptoms was much higher at a mean of 9 points.

Symptom diaries show some advantages over traditional QoL tools. Some of the traditional QoL tools show considerable complexity in their completion. The SGRQ, which is a widely used and validated questionnaire, consists of multiple sections and domains [25]. The QoL-B is more simple and the respiratory symptom domain consists of only 9 questions, but was not designed to detect exacerbations or quantify their severity [20]. Recently developed tools such as the BHQ are also designed to be static measures of disease burden rather than dynamic [21]. The majority of QOL questionnaires have a recall period asking about symptoms over the last week, last month or even 3 months. One of our most striking findings was the large day to day variation in symptoms in many patients even in the absence of an exacerbation. Evidence of such variability suggests there may be a loss in the accuracy of the symptoms that patients report through traditional QoL questionnaires or that patients could find difficulties in deciding which symptoms to report.

One of the most interesting and important findings in our study is around the dynamics of reported exacerbation. We showed that there is often a period of increased symptoms prior to the reporting of an exacerbation. This is important as it suggests a period where exacerbation events may be aborted with appropriate measures. We speculate that such measures might include an increase in the frequency and intensity of airway clearance. Knowledge that there is a pre-exacerbation period where inflammation increases has been used to successfully target anti-inflammatory therapy to prevent exacerbations in asthma [26]. This knowledge is therefore potentially useful in future trials for bronchiectasis. A high variability was observed in the severity and duration of the exacerbations in our cohort of patients with a range of 12–22 points in the symptom score and a range of 5 to 28 days in the duration of the exacerbations respectively. This data is also useful as this cohort collected data on what would be considered “mild” or “moderate” community treated exacerbations and yet the symptoms persisted for more than 2 weeks and in some cases a month. This is important information as the severity and impact of exacerbations is often underestimated. Our study did not include hospitalized severe exacerbations which might be expected to have longer and more severe symptoms.

It has been observed in COPD patients, that failure to report exacerbations is related to higher risk of emergency department and hospital admission, greater lung function deterioration, and worse SGRQ scores than treated exacerbations and thus, may result in a poorer prognosis when compared to treated exacerbations [13–16]. Improved reporting and detection of exacerbations has been achieved in COPD through the use of patient completed diaries. To the authors knowledge our study is the first to characterise unreported and untreated exacerbations in bronchiectasis. Twenty-three unreported exacerbations were noted among 13 patients during the period of study. The peak symptom score during unreported exacerbations was a mean of 11, while the mean duration was 10 days (SD 3.8), showing that unreported exacerbations were milder and shorter than reported ones but nevertheless had an important impact on patients. Although unreported exacerbations appeared to be milder, they may still be important in terms of health repercussion. This was observed in the ATTAIN study where unreported exacerbation in COPD patients had the same medium-term health consequences as reported HCRU exacerbations [27]. Further studies are needed in bronchiectasis patients to discern whether these unreported exacerbations are relevant.

As aforementioned, we detected 23 unreported and 29 reported exacerbations during the study period using the BEST symptom diary. The unreported events represented 44% of the total exacerbations. This suggests the use of symptom diaries in bronchiectasis clinical trials would provide an increased ability to detect both reported and unreported events with a better comprehension of the natural behaviour of the disease, and thereby contribute to an improvement in bronchiectasis research. Many recent bronchiectasis clinical trials have been underpowered due to a failure to capture the expected number of exacerbations during the course of studies [9–12]. There are many reasons for this phenomenon including placebo effects and the Hawthorne effect, but future trials urgently need to take measures to ensure they are adequately powered.

We reported a provisional MCID for the BEST tool in our study based on distribution and anchor based methods. As our sample size is small and the methods used suggested an MCID from 2 to 5 further research is required to establish the true MCID. Nevertheless, our analysis suggests that a change in score of 4 points may be clinically meaningful with a sustained worsening of the score by 4 points or more for 48 h indicative of exacerbation.

The major limitation of our study was the small sample size. Other limitations were the single-centre design of the study and that data collection was conducted for a maximum of 6 months. Diaries place a significant burden on participants by expecting them to complete the information on a daily basis and larger and longer studies would be needed to monitor adherence over for example a 12 month randomized trial. This was a pilot study focused on the development and validation of the tool. Future studies with a large sample size are now required to demonstrate the capability of the tool to improve exacerbation detection, to establish the clinical significance of unreported exacerbations and to understand whether day to day fluctuations in patient symptoms contribute to morbidity and mortality. Incorporation of diaries into clinical trials could provide invaluable information on how therapies affect patients’ symptoms in a far more detailed way than is currently captured.

## Conclusions

The BEST symptom diary shows convergent validity with existing health questionnaires and is responsive at onset and recovery from exacerbation. A daily diary such as BEST may be useful to capture and characterise exacerbations in future trials.

## Supplementary information

**Additional file 1.** Online supplementary material.

## Data Availability

all relevant data is presented in the manuscript and associated figures and tables.

## Abbreviations

6MWD: 6-min walk test
ATTAIN: Safety and tolerability of aclidinium bromide in chronic obstructive pulmonary disease
BEST: Bronchiectasis exacerbation and symptom tool) diary card
BHQ: Bronchiectasis Health Questionnaire
BRR: Bronchiectasis Research Registry
CAT: COPD assessment test
COPD: Chronic obstructive pulmonary disease
CT: Computerized tomography
EMBARC: European Multicentre Bronchiectasis Audit and Research Collaboration
EXACT: Exacerbations of chronic obstructive pulmonary disease tool
FEV_1_: Forced expiratory volume in 1 s
HCRU: Healthcare resources use
LCQ: Leicester cough questionnaire
MICD: Minimum clinically important difference
QoL: Quality of life
QOL-B questionnaire: Quality of life bronchiectasis questionnaire
SGRQ: St George’s Respiratory Questionnaire
