# Supplementary material for: Development and initial validation of the bronchiectasis exacerbation and symptom tool (BEST)
Source: Respir Res. 2020 Jan 13;21:18. doi: 10.1186/s12931-019-1272-y (PMC6958700; doi:10.1186/s12931-019-1272-y)
Supplement: Supplementary file 1 — Additional file 1. Online supplementary material. [file 12931_2019_1272_MOESM1_ESM.docx]

Additional file 1

Development of the BEST diary.

A systematic review of exacerbation definitions used in clinical research from January 2000 until December 2013, involving adults with bronchiectasis not related to CF, was conducted. The search was subsequently updated in December 2015 for the EMBARC/BRR definition of exacerbation project.

Search terms used from a PubMed search were “definition” and “exacerbation” and “exacerbation definition”. Studies related to review articles, descriptive cohorts and editorials were excluded.

The systematic review identified 50 articles with 19 different definitions of exacerbation identified.

| Reference | trial | Center | Country | Definition |
| --- | --- | --- | --- | --- |
| Altenburg J, et al. Jama. 2013;309(12):1251-9. | BAT | Multicenter | Netherlands | Presence of at least 4 of 9 symptoms/signs/findings |
| Bilton et al. Chest. 2013;144(1):215-25 | Efficacy and safety mannitol | Multicenter | UK, Australia, New Zealand | Antibiotic use for flare of disease and pulmonary exacerbation (using a nonvalidated study-designed protocol definition based on the Fuchs definition): Fuchs = presence of 4 of 12 symptoms |
| Bilton et al. Thorax. 2014;69(12):1073-9 | RCT mannitol | Multicenter | UK, US, Belgium, Australia, Netherlands, New Zealand, Argentina, Germany | Worsening of signs and symptoms requiring antibiotic treatment |
| Chalmers et al. Am J Respir Crit Care Med. 2012;186(7):657-65. | short and longterm AB for inflammation | Multicenter | UK | Patient reported exacerbation without further definition though antibiotic use was key |
| Kellett et al. Respir Med. 2011;105(12):1831-5 | Hypertonic saline 7% | Multicenter | UK | Antibiotic use or steroid prescription |
| Mandal et al. Lancet Respir Med. 2014;2(6):455-63. | Atorvastatin | Multicenter | UK | Using "BTS" criteria = start of antibiotic treatment |
| Hernando et al. Int J Clin Pharm. 2012;34(4):644-50. | Budesonide efficacy and safety | Multicenter | Spain | Worsening of at least 3 of 4 symptoms for at least 48h and |
| Lee et al. Respir Res. 2014;15:44 | Exercise training | Multicenter | Australia | Four or more symptoms for more than 48h with OR without antibiotics |
| Barker et al. Lancet Respir Med. 2014;2(9):738-49 | Aztreonam inhalation solution | Multicenter | US, Australia, Spain, Netherlands, UK | acute worsening of respiratory disease meeting at least three major (or two major and at least two minor) criteria. Major criteria were increased sputum production, change in sputum colour, dyspnoea, and cough. Minor criteria were fever (>38°C) at clinic visit, increased malaise or fatigue, FEV1 (L) or forced vital capacity decreased by more than 10% from baseline, and new or increased haemoptysis. |
| Martinez-Garcia et al. Chest. 2012;141(2):461-8. | Budesonide/formoterl | Monocenter | Spain | subjective and persistent (> 24 h) deterioration in at least three respiratory symptoms, including cough, dyspnea, hemoptysis, increased sputum purulence or volume, chest pain (with or without fever), radiographic deterioration, systemic disturbances, or changes in chest auscultation |
| Murray et al. Eur Respir J. 2009;34(5):1086-92. | physiotherapy | Monocenter | UK | An exacerbation was defined as a clinical deterioration with all of the following: increasing cough, increasing sputum volume and worsening sputum purulence. All patients subsequently received antibiotics for 14d |
| Murray et al. 1. Am J Respir Crit Care Med. 2011;183(4):491-9. | Gentamycin | Multicenter | UK | An exacerbation was defined as a clinical deterioration with all of the following: increasing cough, increasing sputum volume and worsening sputum purulence. All patients subsequently received antibiotics for 14d |
| Nicolson et al. Respir Med. 2012;106(5):661-7. | inhaled hypertonic saline 6% | Multicenter | Australia | recording at least three symptoms in one day for two or more consecutive days |
| Patterson et al. Chron Respir Dis. 2007;4(2):67-74. | Acapella vs usual clearance techniques | Monocenter | Northern Ireland | presence of at least 4 of 12 symptoms/signs/findings and antibiotic treatment |
| Serisier et al. Jama. 2013;309(12):1260-7. | BLESS | Multicenter | Australia | antibiotic treatment for a sustained (more than 24h) increase in sputum volume or purulence accompanied by new deteriorations in at least 2 additional symptoms: sputum volume, sputum purulence, cough, dyspnea, chest pain, or hemoptysis. |
| Serisier et al. Thorax. 2013;68(9):812-7. | ORBIT-2 | Multicenter | Australia, UK, New Zealand | was defined as deterioration in at least four of the following nine symptoms or signs: sputum production (volume, colour, consistency or haemoptysis), dyspnoea, cough, lever, wheezing, exercise tolerance (or fatigue/lethargy/ malaise), FEV1 or FVC fall of at least 10%, new changes on chest radiograph and changes in chest sounds on auscultation |
| Valery et al. Lancet Respir Med. 2013;1(8):610-20. | Long-term azithromycin for Indigenous children | Multicenter | Australia, New Zealand | We defined pulmonary exacerbation as treatment by clinic or hospital staff with antibiotics for any of the following (as recorded in the medical chart): increased cough, dyspnoea, increased sputum volume or colour intensity, new chest examination or radiographic findings, deterioration in predicted forced expiratory volume in 1-second (FEV1) percentage by more than 10%, or haemoptysis. |
| Wong et al. Lancet. 2012;380(9842):660-7. | EMBRACE | Multicenter | Australia, New Zealand | TWO definitions: An event-based exacerbation was defined as an increase in or new onset of more than one pulmonary symptom (sputum volume, sputum purulence, or dyspnoea) requiring treatment with antibiotics. For a symptom-based exacerbation to be recorded, the patient had to have an increase in or new onset of more than one pulmonary symptom reported on the daily diary card and the mean of the three symptom scores from the daily diary card on 2 consecutive days had to increase by at least one point (on a five-point scale) compared with the same calculation 1 week earlier. |
| Kapur et al. Pediatr Pulmonol. 2012 Jan;47(1):68-75. | To define NCFB exacerbation in children | Multicenter | Australia | For the study they used a very broad definition as their goal was to "define an exacerbation". Therefore they considered exacerbations as: A sustained worsening of the patient’s condition from stable state and beyond normal day to day variations that is acute in onset and necessitates a change in regular medication. However I will use their results of the study here as exacerbation! Their results show the use of Major, minor and lab results. |

The symptoms most frequently used in prior definitions, and rated by Delphi process as most important were sputum volume, cough, sputum colour, dyspnoea, fatigue and systemic disturbance. Appropriate wording of the diary was modified through feedback with the Tayside patient support group and the European Lung Foundation patient support group who provided input into diary design.
